# Association Between Traffic Count and Cardiovascular Mortality: A Prospective Cohort Study in Taiwan

**DOI:** 10.2188/jea.JE20200082

**Published:** 2021-05-05

**Authors:** Wen-Chi Pan, Szu-Yu Yeh, Chih-Da Wu, Yen-Tsung Huang, Yu-Cheng Chen, Chien-Jen Chen, Hwai-I Yang

**Affiliations:** 1 Institute of Environmental and Occupational Health Sciences, National Yang-Ming University, Taipei, Taiwan; 2 Center of Preventive Medicine, National Yang-Ming University, Taipei, Taiwan; 3 Department of Geomatics, National Cheng Kung University, Tainan, Taiwan; 4 National Health Research Institutes, National Institute of Environmental Health Sciences, Miaoli, Taiwan; 5 Institue of Statistical Science, Academia Sinica, Taipei, Taiwan; 6 National Institution of Environmental Health Sciences, National Health Research Institute, Mioli, Taiwan; 7 Genomics Research Center, Academia Sinica, Taipei, Taiwan; 8 Graduate Institute of Epidemiology and Preventive Medicine, National Taiwan University, Taipei, Taiwan; 9 Institute of Clinical Medicine, National Yang-Ming University, Taipei, Taiwan

**Keywords:** traffic, fine particulate matter, cardiovascular diseases, mediation analysis, Taiwan

## Abstract

**Background:**

Exposure to traffic-related pollution is positively associated with cardiovascular diseases (CVD), but little is known about how different sources of traffic pollution (eg, gasoline-powered cars, diesel-engine vehicles) contribute to CVD. Therefore, we evaluated the association between exposure to different types of engine exhaust and CVD mortality.

**Methods:**

We recruited 12,098 participants from REVEAL-HBV cohort in Taiwan. The CVD mortality in 2000–2014 was ascertained by the Taiwan Death Certificates. Traffic pollution sources (2005–2013) were based on information provided by the Directorate General of Highway in 2005. Exposure to PM_2.5_ was based on a land-use regression model. We applied Cox proportional hazard models to assess the association of traffic vehicle exposure and CVD mortality. A causal mediation analysis was applied to evaluate the mediation effect of PM_2.5_ on the relationship between traffic and CVD mortality.

**Results:**

A total of 382 CVD mortalities were identified from 2000 to 2014. We found participants exposed to higher volumes of small car and truck exhausts had an increased CVD mortality. The adjusted hazard ratio (HR) was 1.10 for small cars (95% confidence interval [CI], 0.94–1.27; *P*-value = 0.23) and 1.24 for truck (95% CI, 1.03–1.51; *P*-value = 0.03) per one unit increment of the logarithm scale. The findings were still robust with further adjustment for different types of vehicles. A causal mediation analysis revealed PM_2.5_ had an over 60% mediation effect on traffic-CVD association.

**Conclusions:**

Exposure to exhaust from trucks or gasoline-powered cars is positively associated with CVD mortality, and air pollution may play a role in this association.

## INTRODUCTION

Cardiovascular disease (CVD) is the leading cause of death globally, and it is estimated that CVD was responsible for more than 30% of global deaths in 2018.^1^ Epidemiological studies suggest long-term exposure to traffic pollution is positively associated with CVD risks.^2^^–^^9^ In a landmark study conducted in the Netherlands, Hoek et al found long-term exposure to traffic-related air pollution is consistently associated with cardiopulmonary mortality.^7^ The findings were robust for various traffic exposure surrogates including black smoke, NO_2_, and living near a major road.

Road-traffic vehicles are generally powered by diesel or gasoline, and evidence has shown diesel engine exhaust (DEE) and gasoline engine exhaust (GEE) may lead to CVD risks. Studies in occupational health settings found that workers chronically exposed to DEE had a higher risk of respiratory symptoms.^10^^–^^12^ Additionally, evidence in human randomized controlled trials indicated short-term exposure to DEE had adverse effects on inflammatory response,^13^^,^^14^ cardiovascular functions,^15^^–^^18^ endothelial functions,^19^ ischemic and thrombotic responses,^20^ and blood pressure.^21^ Nevertheless, previous epidemiological and animal studies suffer from several limitations, and the interpretation of these findings should be viewed with caution.^22^ For instance, participants in most human controlled exposure studies were assigned much higher DEE exposure levels (200 to 300 µg/m^3^) than typical ambient levels.^15^^,^^17^^–^^21^^,^^23^ The unrealistic exposure levels in the experimental setting may not be generalized to the general populations.

Compared to the abundant evidence for DEE exposure and CVD risks, only a limited number of studies evaluated the health impact of GEE, and these were primarily animal studies.^24^^–^^26^ Evidence showed acute exposure to GEE altered cardiac repolarization,^24^ and vascular remodeling pathways involved atherosclerosis.^26^ However, only limited epidemiological studies assessed the health effects of long-term exposure to GEE on CVD risks. Ostro et al found long-term exposure to PM_2.5_ generated from on-road gasoline vehicles was positively associated with risks of ischemic heart diseases, but their findings cannot differentiate the health effect contributed by different type of on-road gasoline vehicles (eg, scooters, gasoline-powered cars).^27^

Given the limitations of previous studies that primarily focused on high level exposure to DEE and the lack of human evidence on GEE exposure, we opted to assess the CVD mortality associated with long-term DEE and GEE exposure in a Taiwanese cohort study. We further elucidated how fine particulate matter (PM_2.5_) mediates the effect of traffic exposure on CVD risks.

## MATERIALS AND METHODS

### Study population and ascertainment of cardiovascular mortality

Initially, we invited 89,293 male and female residents aged 30–65 years who resided on Taiwan’s main island and the Penghu Islets between January 1, 1991 and December 31, 1992. A total of 23,820 participants were successfully recruited with an inform consent in this study (Risk Evaluation of Viral Load Elevation and Associated Liver Disease/Cancer Hepatitis B Virus [REVEAL-HBV]), and details of cohort profile can be found in our previous studies.^28^^–^^31^ Participants residing in the Penghu Islets (*n* = 10,303) were excluded due to the lack of traffic information on major roadways. We further excluded participants missing information for residential address; smoking status; alcohol consumption; serum cholesterol; serum triglyceride; or having a residential address outside of Taipei, Hsinchu, Chiayi, or Pingtung (*n* = 1,419). A total of 12,098 subjects remained for the analysis. Participants were followed through to December 31, 2014. Demographic information and lifestyle factors for each participant were collected using structured questionnaires carried out by public health nurses. A 10 mL peripheral blood sample was fractionated and stored at −70 Celsius for the blood biochemical analysis (eg, serum triglyceride and cholesterol levels). Participants provided written informed consent for the baseline interview, health examinations, biological sample collections, as well as computerized data linkage with the Taiwan Cancer Registry and national death certification system. The diagnosis of CVD events were based on the Taiwan Death Certificate. Participants who died of CVD (ICD-9: 400-440) from Jan 1, 2000 to Dec 31, 2014 were classified as CVD events. All study protocols were approved by the Institutional Review Board of National Yang-Ming University and National Taiwan University.

### Residential exposure to traffic vehicles

Participants’ residential address was geocoded using the web service provided by Taiwan Geospatial One Stop (TGOS), Ministry of Interior (http://tgos.nat.gov.tw/TGOS/Web/TGOS_Home.aspx). The geocoded addresses were transformed using a WGS 84 (World Geodetic 1984) system and stored as a vector-based dataset in ArcGIS 10.1 (ESRI, Redlands, CA, USA). We further extracted the volume of different traffic vehicles (ie, scooter, small car, bus, truck, and semi-trailer) from a database maintained by the Directorate General of Highways, Ministry of Transportation and Communication, Taiwan. We classified scooter and small car as the sources of gasoline exhaust (ie, GEE), whereas bus, truck and semi-trailer contribute to diesel pollution (ie, DEE). A total of 1,318 traffic monitoring sites were included in 2005–2013. The monitoring sites recorded the 3-weekday average traffic volume for each traffic vehicle. We linked each participant’s residential address to the nearest traffic monitoring stations to serve as a surrogate of traffic vehicle exposure in 2005. The median distance of participants’ residential address to the nearest traffic monitoring station was 1.56 km (interquatile range [IQR] = 1.92).

### Exposure assessment of fine particulate matter

The long-term (2005–2014) participants’ residential exposure to PM_2.5_ was primarily based on the land-use regression (LUR) model as we described previously.^32^ In brief, the raw data of PM_2.5_ from 76 stationary monitoring sites were extracted from Taiwan’s Environmental Protection Administration from January 1, 2005 to December 31, 2014. A national land-use inventory, map of industrial park, landmark database, digital road network, Digital Terrain Model, and Moderate Resolution Imaging Spectroradiometer NDVI database were all the potential predictors for PM_2.5_, and were included in the stepwise regression model for the variable selections. *P*-values were 0.1 and 0.3 for the model entry and removal criterion, respectively. The cutoff for the variance inflation factor (VIF) was 3.0 in the final model to avoid the collinearity issue. The R-squared with a 10-fold cross-validation was 0.87, which indicates a sufficient model performance, and the spatial resolution of predicted PM_2.5_ was a regular 250 m × 250 m grid. ArcView GIS (version 10.4) (ESRI Inc.) was applied for the GIS-related calculation.

### Statistical analysis

We analyzed the health impact of the DEE source (bus, truck, and semi-trailer) and GEE source (scooter and small car). A continuous variable of traffic volume (eg, how many trucks per day) was applied throughout the analyses with natural logarithm transformation being performed to stabilize the coefficient estimation. Time at risk for CVD was calculated from the enrollment date to the date of the CVD death (after January 1, 2000) or the last date of follow-up, whichever comes first. The follow-up period (years) was selected as the time scale in the analyses. Cox proportional hazards models were used to estimate the hazard ratios (HRs) and 95% confidence intervals (CIs) for the association between exposure to traffic pollutants and CVD risks. Age (30–39, 40–49, 50–59, or 60–65 years) and sex (male or female) were adjusted in the base model (model 1). Further model adjustment (model 2) on the basis of model 1 was performed for body mass index (BMI; continuous), smoking status (never or ever), serum cholesterol levels (continuous), serum triglyceride levels (continuous), alcohol consumption habit (yes or no), and distance to traffic monitoring sites (continuous). To minimize chance of false positives due to different types of traffic-related pollution, we applied the Benjamini-Hochberg procedure to control multiple comparison issues using false discovery rate <0.2 as the cutoff.

We observed a medium-to-high correlation between different types of vehicles (eFigure 1) and opted to control for the mutual confounding using the ridge regression model (*penalized* packages in R). This approach has been suggested as one of the regression model in multiple exposure setting,^33^^–^^35^ and we previously applied this method to model concurrent exposure to multiple endocrine disruptors in a Taiwanese birth cohort study.^36^ The ridge regression model can simultaneously model high-correlation variables without encountering the collinearity issue, by penalizing the size of the regression coefficients. The choice of the optimal regression coefficients was based on the tuning parameters for penalization selected via 5-fold cross-validation. The corresponding 95 CIs were calculated using 1,000 bootstrap resampling.

A casual mediation analysis was applied to assess the mediation effect of PM_2.5_ on the traffic-CVD association.^37^ We previously utilized this approach to quantify the mediation effect of liver inflammation linking PM_2.5_ exposure and liver cancer.^38^ In brief, we built the mediator model whereby PM_2.5_ was regressed on multiple vehicle exposures and other covariates (ie, distance to traffic monitoring site and participants’ residential county) using a linear regression model. A Cox proportional hazard model was utilized as the outcome model to assess the effect of PM_2.5_ on CVD risks, adjusting for age, sex, smoking status, alcohol consumption, BMI, serum cholesterol levels, serum triglyceride levels, distance to traffic station, residential county, and multiple vehicle exposures (ie, scooter, small car, truck, bus, and semi-trailer). Both the linear regression (mediator model) and Cox proportional hazards models (outcome model) accounted for the collinearity issue of multi-traffic vehicles using the *L2* penalty term (ie, ridge regression). A natural direct effect (NDE) was estimated to quantify the mediation effect of PM2.5 on the traffic-CVD association. Proportion of mediation was derived from the ratio between NDE and total effect (TE).

To test the robustness of our major earlier findings, we restricted the analysis among participants whose CVD events were identified after January 1, 2005 to create a more reasonable temporality between traffic vehicle exposure (2005) and CVD mortality in the follow-up period (2005–2014). All statistical analyses were performed by R statistical program (version 3.5.1; R Foundation for Statistical Computing, Vienna, Austria). A two-side *P*-value of <0.05 was considered statistically significant.

## RESULTS

**Figure 1.**
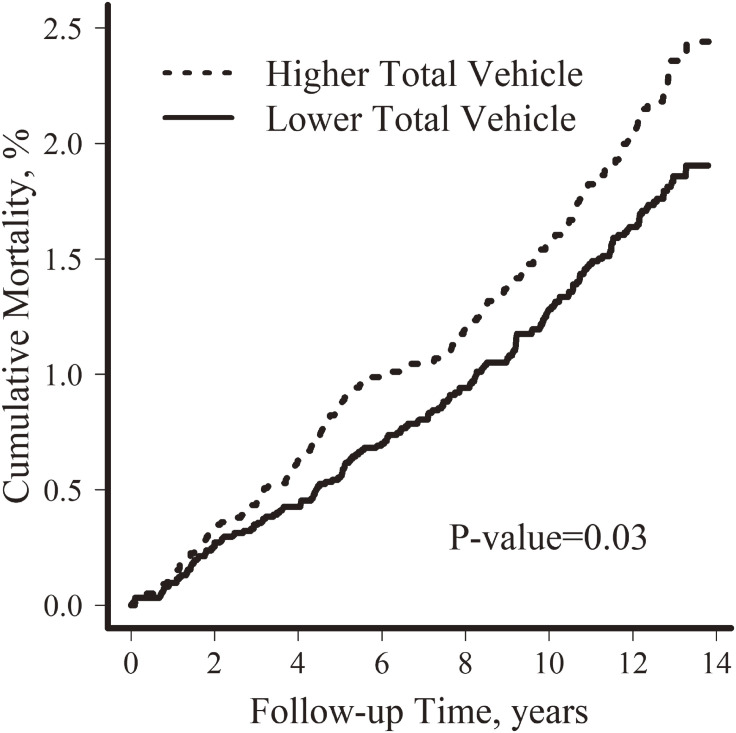
Cumulative mortality of cardiovascular diseases (2000–2014) by total traffic exposure.

A total CVD mortality of 382 was identified with a median follow-up period of 22.9 years. Older age, male sex, higher BMI, had ever smoked, and higher serum triglyceride levels were associated with higher CVD mortality (Table 1). Participants who died from CVD had lower median levels of total vehicle exposure (8,569 [CVD events] versus 8,781 [censored]) (Table 1). After adjusting for age and sex, we found a positive association between total vehicle exposure and cumulative CVD mortality. Participants exposed to higher total traffic (above the median value [5,127 vehicles/day]) had increased CVD mortality compared with the reference group (below median value) (*P*-value = 0.03, Figure 1).

**Table 1.** Baseline characteristics and number of cardiovascular mortality in the REVEAL-HBV cohort

Variable	Censored (*n* = 11,716)		CVD Death (*n* = 382)		*P*-value
	
	*n* (%)	mean (SD)	*n* (%)	mean (SD)	
Age, years					<0.001
30–39	3,347 (28.6)		21 (5.5)		
40–49	3,256 (27.8)		41 (10.7)		
50–59	3,803 (32.5)		181 (47.4)		
60–65	1,310 (11.2)		139 (36.4)		
Sex					<0.001
Female	5,852 (49.9)		139 (36.4)		
Male	5,864 (50.1)		243 (63.6)		
BMI, kg/m^2^		23.9 (3.2)		24.7 (3.3)	<0.001
Ever Smoker					<0.001
No	8,393 (71.6)		212 (55.5)		
Yes	3,323 (28.4)		170 (44.5)		
Alcohol Consumption					0.648
No	10,675 (91.1)		345 (90.3)		
Yes	1,037 (8.9)		37 (9.7)		
Serum Cholesterol, mg/dL		185.9 (42.1)		197.4 (43.5)	<0.001
Serum Triglyceride, mg/dL		133.7 (96.7)		167.4 (108.4)	<0.001
Total Vehicles Exposure, per Day		8,781 (6,474)		8,569 (6,270)	0.156
Scooter		1,595 (1,693)		1,705 (1,645)	0.015
Small Car		6,726 (5,611)		6,434 (5,384)	0.012
Bus		246 (198)		220 (195)	0.004
Truck		101 (62.0)		105 (57.9)	0.097
Semi-trailer		114 (82.6)		104 (82.3)	0.018
PM_2.5_ (2005–2014), µg/m^3^		27.9 (4.93)		28.5 (5.27)	0.024
Distance to Traffic Station, km		1.90 (1.29)		2.01 (1.39)	0.021

CVD, cardiovascular disease; PM_2.5_, fine particulate matter; SD, standard deviation.

To tease out the potential confounders due to other risk factors of CVD mortality, we additionally included BMI, alcohol consumption, serum cholesterol levels, serum triglyceride levels, and distance to traffic station in the regression model. As shown in Table 2, we found increased CVD mortality was associated with higher exposure to small cars, trucks, and total vehicles. The adjusted hazard ratio was 1.10 for small cars (95% CI, 0.94–1.27; *P*-value = 0.23), 1.24 for trucks (95% CI, 1.03–1.51; *P*-value = 0.03), and 1.13 for total vehicles (95% CI, 0.96–1.33; *P*-value = 0.13) with a one-unit increment for vehicle exposure (in natural logarithm scale).

**Table 2.** Association between single traffic exposure and cardiovascular mortality (2000–2014)

Log Traffic Exposure (vehicle/day)	Model 1^a^	*P*-value	Model 2^b^	*P*-value
	
	HR (95% CI)		HR (95% CI)	
Scooter	0.99 (0.95, 1.03)	0.74	1.00 (0.94, 1.04)	0.90
Small Car	1.14 (0.99, 1.32)	0.08	1.10 (0.94, 1.27)	0.23
Bus	0.98 (0.89, 1.08)	0.65	0.95 (0.86, 1.06)	0.36
Truck	1.27 (1.04, 1.53)	0.02^c^	1.24 (1.03, 1.51)	0.03^c^
Semi-trailer	1.00 (0.90, 1.11)	0.98	0.98 (0.88, 1.09)	0.69
Total Vehicle	1.17 (1.01, 1.37)	0.04	1.13 (0.96, 1.33)	0.13

CI, confidence interval; HR, hazard ratio.

^a^Model 1 were adjusted for age and sex.

^b^Model 2 were based on Model 1 with further adjustment for body mass index, smoking, alcohol, serum cholesterol level, serum triglyceride level, and distance to traffic site.

^c^*P*-value with false discovery rate <20% based on Benjamini-Hochberg procedures.

Since different types of vehicles were highly correlated (eFigure 1) and may confound the association between single vehicle exposure and CVD mortality, we further included all types of traffic exposure simultaneously in the ridge regression model to minimize the potential confounding as well as the collinearity issue. We found exposure to small cars was statistically associated with increased CVD mortality. The adjusted HR of small cars was 1.09 (95% CI, 1.01–1.08) per one unit increment in the logarithm scale. A similar trend was also observed for truck exposure (adjusted HR 1.08; 95% CI, 1.00–1.16) (Table 3). We further performed the stratified analysis on sex and found that truck-CVD association was stronger among female participants (HR 1.14; 95% CI, 1.04–1.25) compared to those among male participants (HR 1.04; 95% CI, 0.94–1.11).

**Table 3.** Concurrent exposure to multiple traffic vehicles and its associated cardiovascular mortality (2000–2014)

Log Traffic Exposure (vehicle/day)	HR (95% CI)^a^ among Whole Study	HR (95% CI)^b^ among Male	HR (95% CI)^c^ among Female
Scooter	1.00 (0.96, 1.04)	1.00 (0.96, 1.06)	0.99 (0.94, 1.06)
Small Car	1.09 (1.01, 1.08)	1.06 (0.96, 1.13)	1.08 (0.99, 1.66)
Bus	0.98 (0.94, 1.01)	0.98 (0.90, 1.06)	0.91 (0.82, 1.01)
Truck	1.08 (1.00, 1.16)	1.04 (0.94, 1.11)	1.14 (1.04, 1.25)
Semi-trailer	0.99 (0.91, 1.07)	1.02 (0.92, 1.10)	0.96 (0.87, 1.07)

CI, confidence interval; HR, hazard ratio.

^a^Model was adjusted for age, sex, smoking, alcohol, body mass index (BMI), serum cholesterol levels, serum triglyceride levels, distance to station (log-scale), and multiple traffic vehicles.

^b^Model was adjusted for age, smoking, alcohol, BMI, serum cholesterol levels, serum triglyceride levels, distance to station (log-scale), and multiple traffic vehicles.

^c^Model was adjusted for age, BMI, serum cholesterol levels, serum triglyceride levels, distance to station (log-scale), and multiple traffic vehicles.

Table 4 estimated the effect of traffic exposure (ie, small cars and trucks) on CVD death that was mediated through PM_2.5_. The natural indirect effects of PM_2.5_ was positive for exposure to small cars (adjusted HR 1.05; 95% CI, 1.01–1.09) and trucks (adjusted HR 1.08; 95% CI, 1.02–1.15) in terms of CVD mortality, and showing statistical significance. Additionally, the mediation proportions were 0.63 for small cars and 0.85 for trucks.

**Table 4.** Mediation effect of fine particulate matter on traffic-associated mortality of cardiovascular diseases (2000–2014)

Log Traffic Exposure (vehicle/day)	HR (95% CI)^a^	Proportion of Mediation
Small Car	1.05 (1.01, 1.09)	0.63
Truck	1.08 (1.02, 1.15)	0.85

CI, confidence interval; HR, hazard ratio.

^a^Mediator Models were adjusted for distance to traffic station, participants’ residential county, and multiple traffic vehicles. Outcome models were adjusted for age, sex, smoking, alcohol consumption, body mass index, serum cholesterol, serum triglyceride, distance to traffic station, residential county, and multiple traffic vehicles.

In sensitivity analysis, we excluded participants who had CVD events prior to January 1, 2005, and it created a better temporality between traffic vehicle exposure (2005) and CVD mortality afterward (2005–2014). Similarly, truck exposure was positively associated with CVD mortality (adjusted HR 1.19; 95% CI, 0.95–1.50) in a single traffic exposure setting (eTable 1), and further adjustment for concurrent exposure to other traffic vehicles did not change the directionality (adjusted HR 1.09; 95% CI, 0.99–1.21) (eTable 2). Sensitivity findings in small cars showed weaker association with CVD mortality (adjusted HR 1.03) with wider 95% CI (0.86–1.23) (eTable 1) compared with those in the main analysis (adjusted HR 1.10; 95% CI, 0.94–1.27) (Table 2). Mediation analysis shown in the supplementary materials (eTable 3) revealed 66% of small car-associated CVD mortality was mediated by PM_2.5_, whereas this number was higher for truck exposure (80%).

## DISCUSSION

This community-based cohort study found exposure to the exhaust of small cars or trucks was associated to a statistically significant level with an increased risk of CVD mortality, controlling for concurrent exposure to different types of vehicles. This finding suggested not only diesel-engine pollution can trigger CVD, but also exhaust derived from gasoline-powered vehicles may lead to cardiovascular mortality. The mediation analysis showed that PM_2.5_ was the major route linking traffic exposure and CVD death. This may be among the first pieces of epidemiological evidence to demonstrate the potential CVD risks of exposure to pollutants generated by gasoline-powered vehicles.

A study based on Netherland Cohort Study on Diet and Cancer found traffic intensity (per 10,000 motor vehicles/24 hours) on the nearest roadway of participants’ residential address was positively associated with ischemic heart diseases (HR 1.11; 95 CI, 1.03–1.20), and the results were robust following the adjustment for air pollution indicators and traffic noise.^39^ Beelen et al used traffic volume (ie, numbers of total motor vehicles per 24 hours) instead of a specific exposure to vehicle type (eg, small cars, trucks) as the traffic exposure index, and their findings were consistent with the results shown in this study where we found a positive association between total vehicle exposure and CVD mortality (HR 1.13; 95% CI=, 0.96–1.33) (Table 2). A more recent study conducted in London, United Kingdom examined the relationship between CVD/respiratory hospital admissions and short-term exposure to various sources of traffic pollution.^40^ Their findings showed short-term exposure to diesel vehicle exhaust was consistently associated with adult cardiovascular events by using elementary carbon in PM_10_ and black carbon in PM_2.5_ as exposure indicators. However, short-term exposure to general traffic (NO_x_ as indicator) and petrol vehicle exhaust (CO as indicator) showed weaker associations.

Accumulative human studies indicate exposure to DEE impairs cardiovascular and respiratory function. Evidence based on the occupational population shows workers chronically exposed to DEE had a higher risk of respiratory symptoms.^10^^–^^12^ Recent studies that recruited volunteer human subjects indicated short-term exposure to DEE had adverse cardiovascular effects.^13^^–^^17^^,^^19^^–^^21^ A 1-hour DEE chamber exposure with a concentration of 300 µg/m^3^ increased ischemic burden and inhibited endogenous fibrinolytic capacity among male participants.^20^ In a randomized double-blind crossover study among healthy non-smokers, participants exposed to diesel exhaust generated by a diesel engine had higher basal concentration of NO release that may have led to abnormal vascular homeostasis.^17^ Additionally, a recent epidemiological study conducted in Japan demonstrated the relationship between long-term exposure to DEE and CVD mortality.^41^ A quasi-experiment study conducted in Tokyo, Japan evaluated how much mortality burden can be reduced by diesel emission control ordinance introduced in 2003. The results showed that introducing the control program significantly reduced mortality specific to cardiopulmonary disease and lung cancer, along with reduced levels of PM_2.5_.

The evidence to elucidate the cardiovascular health effect of GEE exposure was primarily based on animal studies, and no human evidence has been ever reported. Lund et al found 7-week exposure to GEE (8 to 60 µg/m^3^) increased gene expression related to vascular remodeling and oxidative stress in apolipoprotein E-deficient mice, indicating GEE exposure may facilitate the progression of atherosclerosis and make vulnerable plagues unstable.^26^ Using a similar experiment setting in Apolipoprotein E (ApoE−/−) mice, the evidence showed exposure to GEE for 60 consecutive days (6 hour per day) had altered levels of endothelia factors (VEGF, ET-1), oxidative stress markers (HO-1, TBARS), and matrix metalloproteinase (MMP-7, MMP-9).^42^ An additional animal study demonstrated there was a significant deviation in T-wave and increased levels of plasma endothlin-1 among mice exposed to GEE for 3 days, whereas paved road dust had no effect.^24^

The interpretation of this study’s findings should be treated with caution given several inherent limitations. First, a 1-year traffic exposure (2005) was used to represent the long-term exposure to GEE or DEE, and this may have introduced certain levels of exposure misclassification. However, the 1-year (2005) and 9-year (2005–2013) traffic information was highly correlated, and Spearman’s correlation was greater than 0.7 for all types of vehicle, except for trucks (eTable 4). Second, we applied a 10-year mean value of PM_2.5_ between 2005 and 2014 that may not perfectly reflect participants’ long-term exposure to air pollution. As discussed by previous studies,^43^^–^^45^ several years of PM_2.5_ levels could serve as a surrogate for decade-long exposure if annual exposure is highly correlated and uniform exposure ranking holds over time by geographical location. We tested whether the above assumptions using PM_10_ information can be tracked from 1994 to 2014, and the results did not violate these assumptions. Third, since we could not retrieve participants’ CVD inpatient and outpatient records, we opted to use CVD mortality confirmed using death certificates as cardiovascular events. This raises the issue that participants who had CVD incidence may not totally be identified using a death certificate, and we would underestimate the cardiovascular burden in this study. Fourth, we did not adjust for some risk factors of CVD (eg, blood pressure, type 2 diabetes status) due to lack of information at baseline, and it may introduce the unmeasured confounding. Nevertheless, traffic-CVD association was adjusted for serum cholesterol and triglyceride levels that may partially correct this bias. Fifth, although our study findings suggested that the traffic-CVD association was partially mediated by PM_2.5_, noise information that was not available in this study could be another factor linking traffic exposure and CVD mortality. Last, the estimation of participants’ traffic exposure was based on the nearest monitoring station whereas the level of participants’ long-term PM_2.5_ exposure was estimated using a LUR approach with a finer spatial resolution (ie, 250 m × 250 m grid). It may result in a more accurate estimation for the PM_2.5_-CVD association compared with the traffic-CVD relationship.

There are certain strengths to this study. We collected different types of vehicle information (ie, gasoline-powered cars, diesel-engine vehicles) and examined their association with participants’ CVD risks. This could clarify the different health impacts of GEE and DEE exposure. Further, by introducing the ridge regression models in the analysis, we could minimize the collinearity issue as well as mutual confounding of concurrent exposure to multiple vehicles that was rarely considered in previous studies.

### Conclusions

To conclude, we found exposure to engine exhaust generated from specific types of gasoline-powered (ie, small cars) or diesel-engine vehicles (ie, trucks) was positively associated with cardiovascular mortality, and PM_2.5_ was the major mediator linking traffic exposure and CVD risk. More observational studies should be conducted to externally validate our findings. Further, new evidence based on toxicological experiments will enlighten our understanding of how gasoline-derived pollutants trigger adverse cardiovascular responses.

## APPENDIX A. SUPPLEMENTARY DATA

eTable 1. Correlation between traffic vehicle exposure information in 2005 and 2005–2013

eTable 2. Association between single traffic exposure and cardiovascular mortality (2005–2014)

eTable 3. Concurrent exposure to multiple traffic vehicles and its associated cardiovascular mortality (2005–2014)

eTable 4. Mediation effect of fine particulate matter on traffic-associated mortality of cardiovascular diseases (2005–2014)

eFigure 1. Correlation matrix between traffic vehicle exposures in 2005
