# Supplementary material for: Association Between Traffic Count and Cardiovascular Mortality: A Prospective Cohort Study in Taiwan
Source: J Epidemiol. 2021 May 5;31(5):343–9. doi: 10.2188/jea.JE20200082 (PMC8021879; doi:10.2188/jea.JE20200082)

Supplementary Materials

Association between Traffic Count and Cardiovascular Mortality: A Prospective Cohort Study in Taiwan

Wen-Chi Pan, [1,2*] Szu-Yu Yeh,[1,2] Chih-Da Wu, [3,4] Yen-Tsung Huang[5], Yu-Chen Chen,[6] Chien-Jen Chen, [7,8] and Hwai-I Yang[7,9*]

[1]Institute of Environmental and Occupational Health Sciences, National Yang-Ming University, Taipei, Taiwan

[2]Center of Preventive Medicine, National Yang-Ming University, Taipei, Taiwan

[3]Department of Geomatics, National Cheng Kung University, Tainan, Taiwan

[4]National Health Research Institutes, National Institute of Environmental Health Sciences, Miaoli, Taiwan

[5]Institue of Statistical Science, Academia Sinica, Taipei, Taiwan

[6]National Institution of Environmental Health Sciences, National Health Research Institute, Mioli, Taiwan

[7]Genomics Research Center, Academia Sinica, Taipei, Taiwan

[8]Graduate Institute of Epidemiology and Preventive Medicine, National Taiwan University, Taipei, Taiwan

[9]Institute of Clinical Medicine, National Yang-Ming University, Taipei, Taiwan

eTable 1. Association between single traffic exposure and cardiovascular mortality (2005–2014)

	Model 1[a]		Model 2[b]	
Log Traffic Exposure (vehicle/day)	HR (95% CI)	P-value	HR (95% CI)	P-value
Scooter	1.00 (0.96, 1.06)	0.85	1.01 (0.97, 1.06)	0.59
Small Car	1.06 (0.89, 1.26)	0.52	1.03 (0.86, 1.23)	0.78
Bus	0.85 (0.85, 1.07)	0.38	0.93 (0.82, 1.05)	0.22
Truck	1.20 (0.96, 1.51)	0.11	1.19 (0.95, 1.50)	0.13
Semi-trailer	1.00 (0.89, 1.13)	0.98	0.98 (0.86, 1.11)	0.71
Total Vehicle	1.09(0.90, 1.31)	0.39	1.05 (0.87, 1.28)	0.59

CI, confidence interval; HR, hazard ratio.

[a]Model 1 was adjusted for age and sex.

[b]Model 2 was based on Model 1 with further adjustment for body mass index, smoking, alcohol, cholesterol, triglyceride, and distance to traffic site

eTable 2. Concurrent exposure to multiple traffic vehicles and its associated cardiovascular mortality (2005–2014)

Log Traffic Exposure (vehicle/day)	HR(95% CI)[a]
Scooter	1.00 (0.96, 1.04)
Small Car	1.11 (1.00, 1.21)
Bus	0.93 (0.83, 1.03)
Truck	1.09 (0.99, 1.21)
Semi-trailer	1.00 (0.88, 1.13)

CI, confidence interval; HR, hazard ratio.

[a]Model was adjusted for age, sex, smoking, alcohol, body mass index, serum cholesterol levels, serum triglyceride levels, distance to station (log-scale), and multiple traffic vehicles.

eTable 3. Mediation effect of fine particulate matter on traffic-associated mortality of cardiovascular diseases (2005–2014)

Traffic, log (vehicle/day)	HR(95% CI)[a]	Proportion of Mediation
Small Car	1.06 (1.02, 1.10)	0.66
Truck	1.08 (1.03, 1.16)	0.80

CI, confidence interval; HR, hazard ratio.

[a] Mediator Models were adjusted for distance to traffic station, participants' residential county, and multiple traffic vehicles. Outcome models were adjusted for age, sex, smoking, alcohol consumption, body mass index, serum cholesterol, serum triglyceride, distance to traffic station, residential county, and multiple traffic vehicles.

eTable 4. Correlation between traffic vehicle exposure information in 2005 and 2005–2013

Traffic Vehicle Exposure	Spearman Correlation Coefficient between 2005 and 2005–2013 (mean) measurements
Scooter	0.85
Small Car	0.98
Bus	0.76
Truck	-0.07
Semi-trailer	0.77
Total Vehicles	0.96

eFigure 1. Correlation matrix between traffic vehicle exposures in 2005

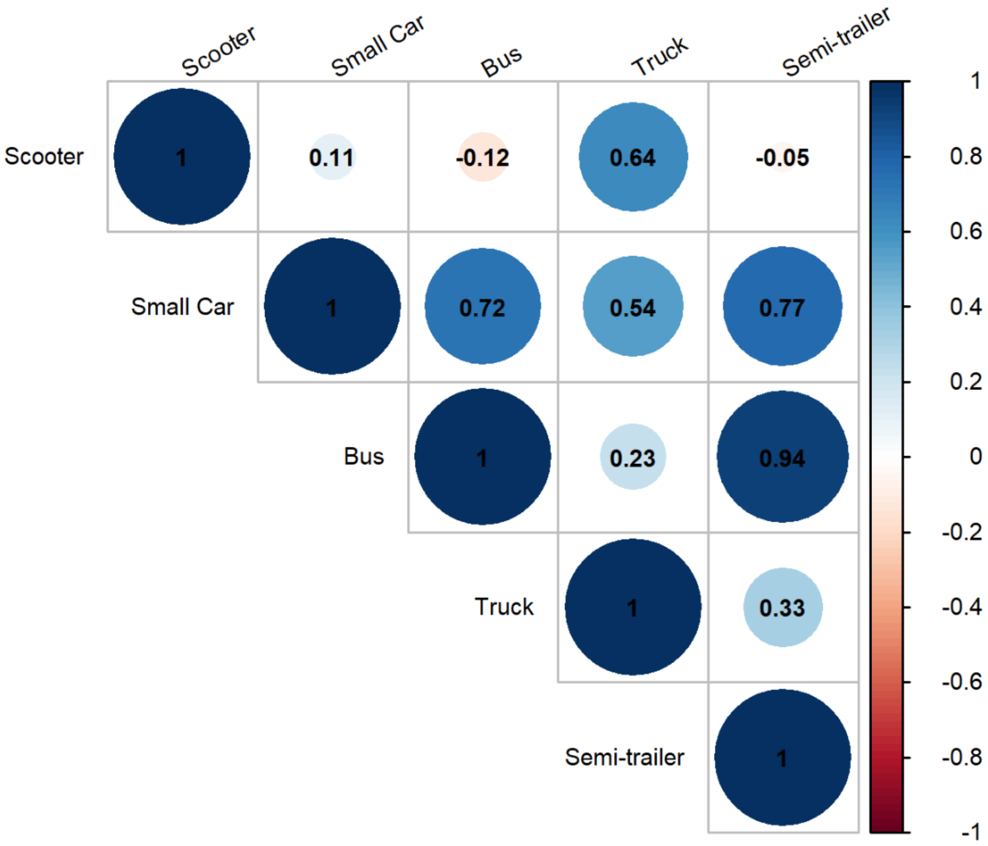

Supplement: Supplementary file 1 [file je-31-343-s001.pdf]
